# Genome-Wide Identification and Analysis of Polygalacturonase Genes in *Solanum lycopersicum*

**DOI:** 10.3390/ijms19082290

**Published:** 2018-08-04

**Authors:** Xubo Ke, Huasen Wang, Yang Li, Biao Zhu, Yunxiang Zang, Yong He, Jiashu Cao, Zhujun Zhu, Youjian Yu

**Affiliations:** 1 Key Laboratory for Quality Improvement of Agricultural Products of Zhejiang Province, College of Agricultural and Food Science, Zhejiang A&F University, Wusu Street 666, Lin’an, Hangzhou 311300, China; KXB2285@163.com (X.K.); whsych66@163.com (H.W.); lylily0327@163.com (Y.L.); billzhu@zafu.edu.cn (B.Z.); yxzang78@163.com (Y.Z.); heyong@zafu.edu.cn (Y.H.); 2 Laboratory of Cell & Molecular Biology, Institute of Vegetable Science, Zhejiang University, Hangzhou 310058, China; jshcao@zju.edu.cn

**Keywords:** tomato, *Solanum lycopersicum*, polygalacturonase, gene family, phylogenetic analysis, expression analysis

## Abstract

Polygalacturonase (PG), a large hydrolase family in plants, is involved in pectin disassembly of the cell wall in plants. The present study aims to characterize PG genes and investigate their expression patterns in *Solanum lycopersicum*. We identified 54 PG genes in the tomato genome and compared their amino acid sequences with their Arabidopsis counterpart. Subsequently, we renamed these PG genes according to their Arabidopsis homologs. Phylogenetic and evolutionary analysis revealed that these tomato PG genes could be classified into seven clades, and within each clade the exon/intron structures were conserved. Expression profiles analysis through quantitive real-time polymerase chain reaction (qRT-PCR) revealed that most *SlPGs* had specific or high expression patterns in at least one organ, and particularly five PG genes (*SlPG14*, *SlPG15*, *SlPG49*, *SlPG70*, and *SlPG71*) associated with fruit development. Promoter analysis showed that more than three *cis*-elements associated with plant hormone response, environmental stress response or specific organ/tissue development exhibited in each SlPG promoter regions. In conclusion, our results may provide new insights for the further study of PG gene function during plant development.

## 1. Introduction

Polygalacturonases (PGs) is an enzyme catalyzing the hydrolysis and disassembly of pectin, a major component of the cell wall in plants [1]. Generally, the pectin network in cell walls needs to be disassembled along with the plant cells undergoing changes in shape. Therefore, PGs are indispensable for almost all stages of plant development, such as organ shedding, fruit ripening, anther dehiscence, and pollen ripening [2,3,4]. Based on the different catalyzing processes, PGs could be mainly classified into three groups: endo-PGs, exo-PGs, and rhamno-PGs. They are associated with many cell-separation processes in plant development as determined by many isolation and characterization analyses [5,6]. For instance, Ogawa et al. [4] have reported that the knockout of two PG genes of Arabidopsis (*AtQRT2* and *AtQRT3*) resulted in the generation of tetrad pollen due to the failure of degradation of pectin in the pollen mother cell wall during the tetrad stage.

Studies have reported that PGs are encoded by a large gene family, and the evolution of plant PG gene family has been explored by many researchers [7]. Up to now, the expression patterns of 75, 53, 46, 68, 99, and 85 PG genes in *Populus*, *Cucumis sativus*, *Oryza sativa*, *Arabidopsis thaliana*, *Brassica rapa* and *Malus × domestica* have been respectively investigated [8,9,10,11,12]. Additionally, a total of 577 PGs have been identified from five grasses and five dicots. In addition, the evolution, expression, and *cis*-regulatory element of them were comprehensively compared [7]. Although most PG genes function in vegetative growth, there are some fruit-specific PG genes acting on the development and maturation of the fruit as well as the formation of abscission zone in fruit stalks after fruit ripening [2,13]. To date, PG genes have been widely studied in the fruits of many species, such as apple (*MdPG36*), banana (*MAPG1* to *MAPG4*), pear (*PcPGl* and *PcPG3*), and grape (*VvPG1* and *VvPG2*) [11,14,15,16,17]. Understanding the function and regulatory mechanism of PG genes associated with fruit development is of great interest for fruit production.

In the process of fruit growth and development, there are not only changes in morphology and structure, but also complex physiological and biochemical changes, including the changes of aroma, flavor, color, texture, and containing substances during fruit ripening. PGs can catalyze the cleavage of α-(1,4)-galacturonic acid in pectin molecules, involved in the degradation of pectin to promote fruit ripening and softening [18]. Hadfield and Bennett [2] have reported that the fruit ripening process is accompanied by the pectin degradation, and the increase of soluble pectin and pectic acid, PG activity, and pore size of the cell wall, as well as fruit softening. Furthermore, the contact with the substrate accelerates cell disintegration, eventually leading to morphological changes in the process of fruit ripening. Hobson [19] first demonstrated that in mature green tomatoes there was no extractable PG activity, while the enzyme appeared at the commencement of coloration and then increased dramatically, suggesting that the increased PG activity was closely related to fruit ripening and softening. Subsequently, Tucker et al. [20] suggested that the activity arose from two isoenzymes (PG1 and PG2) which sequentially appeared during ripening. PG is also the first hydrolase that is examined with transgenic methods in tomato [21]. However, some previous transgenic studies found that PG gene may be unnecessary for the maturation and softening of tomato fruit [22,23,24]. Therefore, it was hypothesized that PG-mediated pectin disassembly during ripening makes only a small contribution to fruit softening. Recently, studies on apple, papaya, strawberry, and tomato suggest a critical role for pectin modifications in fruit softening, which challenges the hypothesis above [25,26,27,28]. Taken together, different PG genes may have divergence in temporal and spatial expression during the long period of fruit development. Currently, the PG genes associated with fruit ripening have not been thoroughly screened out in tomato.

Therefore, in this study, we intended to trace all the members of the PG gene family in tomatoes and to analyze their genomic structures, chromosomal locations, protein conserved domains, evolutionary relationship, and *cis*-elements of their promoters. A phylogenetic analysis using PG genes from Arabidopsis and *S. lycopersicum* was also performed. In addition, the expression level of all SlPG genes in different organs and during fruit development of tomato was quantitatively analyzed. The data from this study will provide useful information for the future study of biological functions of PGs in tomato and other Solanaceae crops.

## 2. Results

### 2.1. Identification and Genomic Distribution of Tomato PG Genes

A total of 54 PG gene sequences were retrieved from the tomato genome for members of tomato PG gene family based on the TBLASTN (search translated nucleotide databases using a protein query and the basic local alignment search tool) search against the tomato genome database (available online: http://solgenomics.net/) (Table 1 and Table S1). The open reading frame (ORF) was from 1161 bp (*SlPG37-3*, *SlPG24-1*, and *SlPG24-4*) to 1524 bp (*SlPG9*) in length. The coding protein ranged from 387 to 508 amino acids with a molecular mass of 41.31 kDa (SlPG24-1) to 55.67 kDa (SlPG9). The isoelectric point ranged from 4.88 (SlPG6) to 9.77 (SlPG21-1). Additionally, among the 54 tomato PGs, 38 have possible signal peptide sequences, the lengths of which ranged from 17 aa (SlPG24-5, etc.) to 31 aa (SlPG56-2, etc.) (Table 1). The predicted subcellular localization of most SlPGs was located in the secretory pathway, except for SlPG57 and SlPG58-1 (mitochondrial) as well as SlPG6 (chloroplast) (Table 1).

### 2.2. Sequence Analysis and Genomic Distribution of Tomato PG Genes

**Figure 1:**
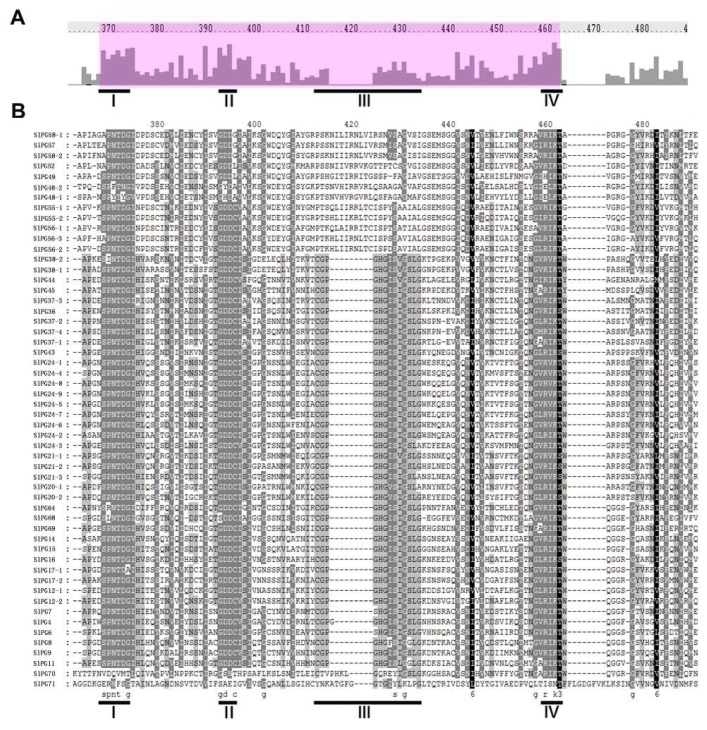
Multiple sequence alignment analysis of the peptides of polygalacturonases (PGs) containing the four typical conserved domains in *S. lycopersicum*. (**A**) Alignment of the tomato PG peptides containing the four typical conserved domains. The height of the bars indicates the number of identical residues per position. The underlines illustrate the four typical conserved domains of PGs; (**B**) Multiple alignments of the peptides of tomato PGs containing four conserved domains. Black and light gray shading, respectively, indicate identical and conserved amino acid residues. Conserved domains are also underlined and correspond to part A.

**Figure 2:**
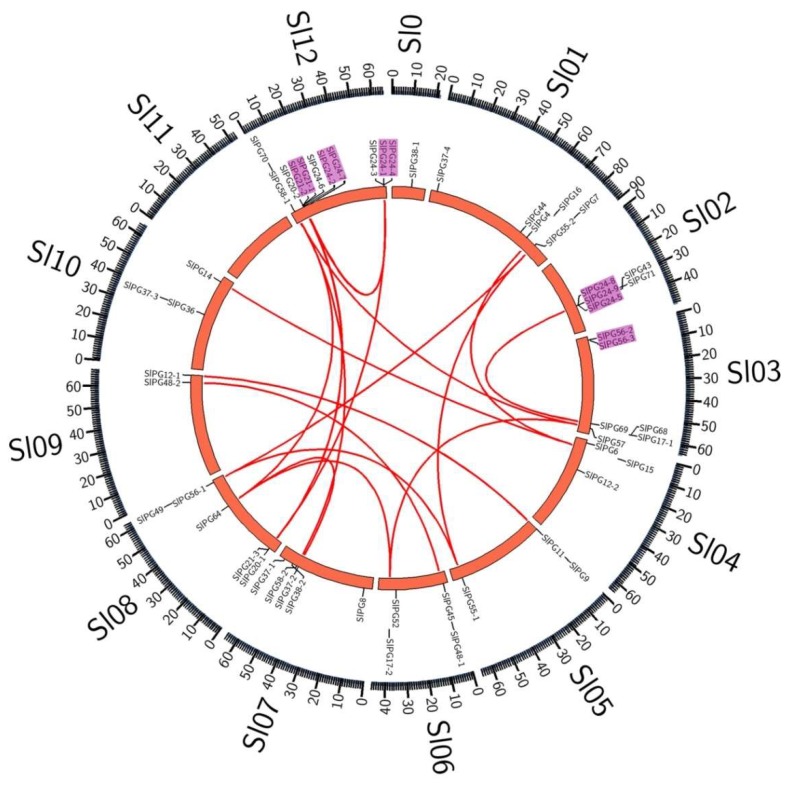
Genomic distribution and syntenic analysis of PG genes in *S. lycopersicum* generated by CoGe comparative genomics system [31] and Circos [32]. The red lines represent syntenic relationships of the paralogous SlPG genes. The chromosome numbers are demonstrated next to each chromosome. Tandem-duplicated genes are indicated by the pink rectangles.

Four conserved domains (motif I to IV) hypothesized to be essential for PG hydrolysis activity have been identified in most PG members from different species [29]. Using the Clustal X software, multiple-sequence alignment and conserved domains analysis of the SlPG sequences were performed. Among the 54 PGs, 40 had four typical conserved domains, 12 did not contain the third conserved domain, and one lacked the first three domains (Figure 1 and Table 1). As a well-known PG, AtQRT3 has been proved to participate in pectin degradation of the pollen mother cell wall during the tetrad stage of pollen development [30]. None of the four domains were present in the protein sequences encoded by the homologous gene (*SlPG71*) of *AtQRT3* in *S. lycopersicum* (Figure 1 and Table 1). Chromosome mapping of the tomato PG gene was not randomly distributed on 11 of the 12 chromosomes in the genome (Figure 2). One gene was found on chromosome 0; two were found on chromosome 9; three were found on chromosomes 4, 5, and 10; four were found on chromosome 6; five were found on chromosomes 2, 7, and 8; six were found on chromosome 1 and 3, and 11 were found on chromosome 12. There were 28 tomato PG genes distributed on the chromosomes, which were clustered into 10 clusters, including 5 tandem repeats gene clusters (Figure 2). The synteny comparison between the paralogous gene pairs revealed that 25 sets of SlPG genes were observed in tomato genomes.

### 2.3. Phylogenetic and Gene Structure Analysis

**Figure 3:**
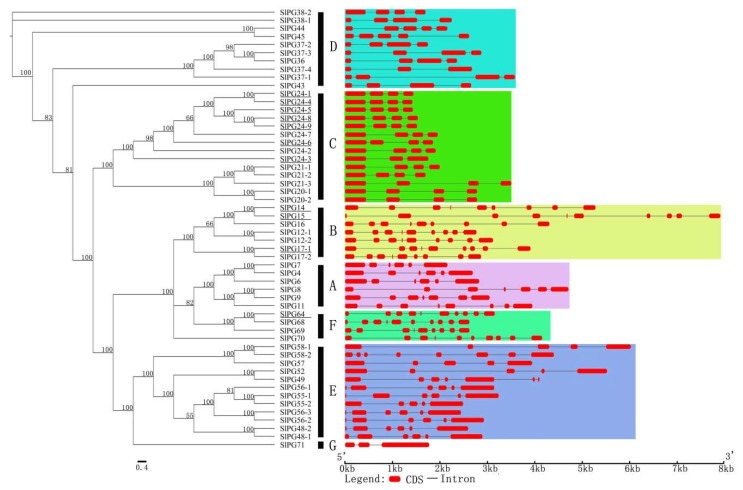
Phylogenetic analysis and intron/exon organization of tomato PG genes. Left part indicates the phylogenetic tree of tomato PG genes constructed based on the amino acid sequences. Different clades are named for their closest corresponding clades in previous studies on the evolution of PGs [12]. The SlPGs that have been reported by previous studies are indicated with underlines. Right part illustrates the intron/exon configurations of the corresponding tomato PG genes. The red rounded rectangle indicates the exon, and the line indicates the intron. Gene structures of PG genes in different clades are shaded by different colors.

A rooted phylogenetic tree including 54 SlPGs was obtained with the Bayesian inference (BI) method. The tree was divided into seven main clades (Clade A to G) (Figure 3). Clade G was composed of *SlPG71* encoded by the homologous gene of *AtQRT3* in *S. lycopersicum*. Based on the phylogenetic tree, the exon/intron structures of the 54 SlPG genes were analyzed by Gene Structure Display Server (GSDS) with their full-length coding sequences and corresponding genomic DNA sequences (Table S1). The results revealed that the number of SlPG introns ranged from 2 (*SlPG37-4*, *SlPG24-3*, and *SlPG71*) to 9 (*SlPG15*, *SlPG64*, *SlPG68*, and *SlPG70*). *SlPGs* of clades A, B, and F contained relatively more introns than those of other clades. In addition, the gene structures and intron lengths were relatively conserved among the members within the same clade, while they were much different between the members of different clades such as clade B and C.

To further understand the evolutionary relationship of these PG genes, 68 PG sequences of Arabidopsis obtained from the TAIR database, and 54 SlPGs were used to generate another rooted joint phylogenetic tree (Figure S1). Seven clades were observed, which were named as Clade A to G as well. Additionally, they could be further divided into 20 sub-clades. A large number of sub-clade members, such as CI and DIII, were mostly composed of PG genes of Arabidopsis or *S. lycopersicum*, and these sub-clades were basically distributed in C, D, and F PG genes. Members of sub-clade FIc were all composed of PG genes in Arabidopsis. Based on the phylogenetic analysis, we first named the 54 SlPGs according to the systematically named Arabidopsis PGs (Figure S1, Table S2). In addition, combined with the syntenic analysis of *SlPGs,* two conserved syntenic blocks with different pairs of duplicated PG genes were found to present inside the tomato genomes (*SlPG24-2*/*24-7* and *SlPG24-1*/*24-4*; *SlPG20-1*/*21-3* and *SlPG20-2*/*21-1*/*21-2*) (Figure 2 and Figure 3).

### 2.4. Expression Patterns of SlPG Genes

**Figure 4:**
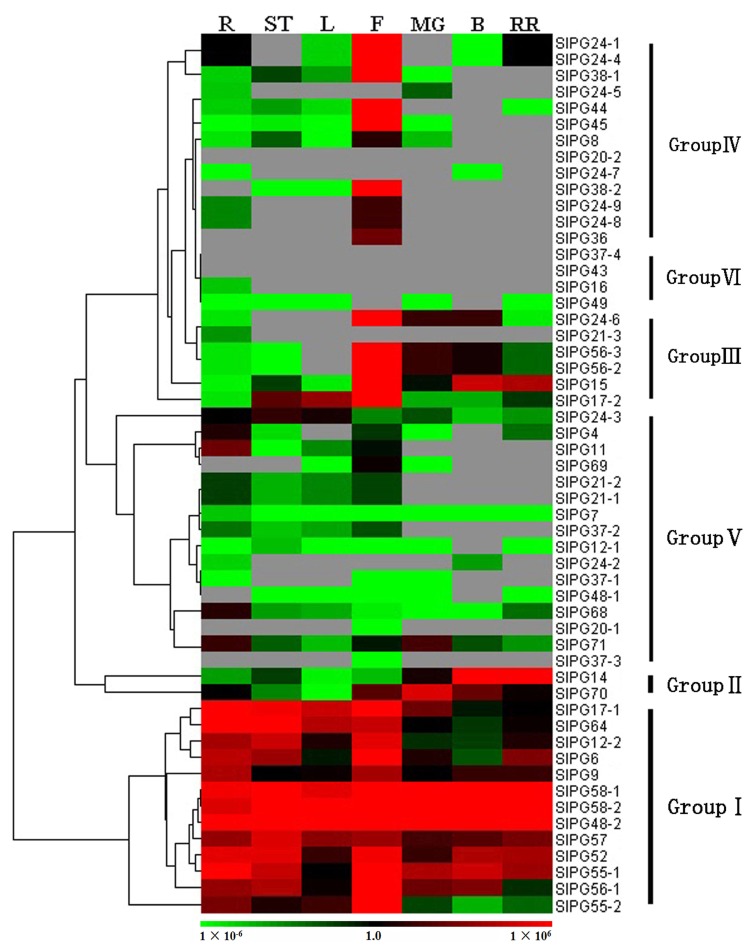
Hierarchical clustering and heat map generated by Cluster 3.0 showing the expression levels of SlPG genes among different tissues of *S. lycopersicum*. The scale bars represent relative expression level. The bright green, bright red, and black shading indicate relatively low, high, and medium expression respectively. The grey shading designates undetectable expression. The vertical dark bar on the right illustrates the six groups of SlPG genes. Different groups are named according to the method used in previous studies [12]. R: roots, ST: stems, L: leaves, F: flowers, MG: mature green fruit, B: breaker fruit, RR: red ripening fruit.

The expression patterns of all *SlPGs* in roots, leaves, stems, flowers, and the fruits of three different development stages (mature green, breaker, and red ripening stage) were determined by quantitive real-time polymerase chain reaction (qRT-PCR). As shown in Figure 4, 51 of the 54 SlPG genes were expressed in at least one organ. The 54 PG genes were divided into 6 groups (Group I to VI) according to their tissue specificity and relative gene expression levels as illustrated by Cluster 3.0. Group I contained 13 genes, whose expression could be detected in all organs without significant difference. Genes in Group II exhibited the highest expression levels in fruits, and in Group III and Group IV (except for *SlPG21-3*, *SlPG20-2*, *SlPG24-5*, and *SlPG24-7*) showed the highest expression levels in flowers. Genes in Group V and Group VI were not detected or had very low expression levels in the seven tomato tissues. Most of the genes in Clade E were clustered into Group I (61.5%) the members of which tended to be ubiquitously expressed (Table S3). Most of the genes in Clade C, D, and F belonged to Group IV and Group V, indicating high or specific expression in flowers or low expression levels in all tissues. Members of Clade A and Clade B were clustered into 3 and 5 Groups, respectively. Specifically, in tomato PG gene family, one member in Clade C (*SlPG20-2*) and two in Clade D (*SlPG37-4*, *SlPG43*) failed to be detected by qRT-PCR in our study.

To confirm the expression patterns of SlPG genes in different organs/tissues of tomato, we further investigated their expression profiles using the available RNA-seq-based datasets of Tomato eFP Browser (TEB) [33]. The results showed that the expression patterns of most SlPG genes among the ten different tomato main organs were consistent with our findings (Figure S2). In particular, *SlPG20-2* failed to be detected by our qRT-PCR analysis and was found to have a low and specific expression in roots.

### 2.5. Specific Expression of PG Genes in Three Developmental Stages of Tomato Fruit

**Figure 5:**
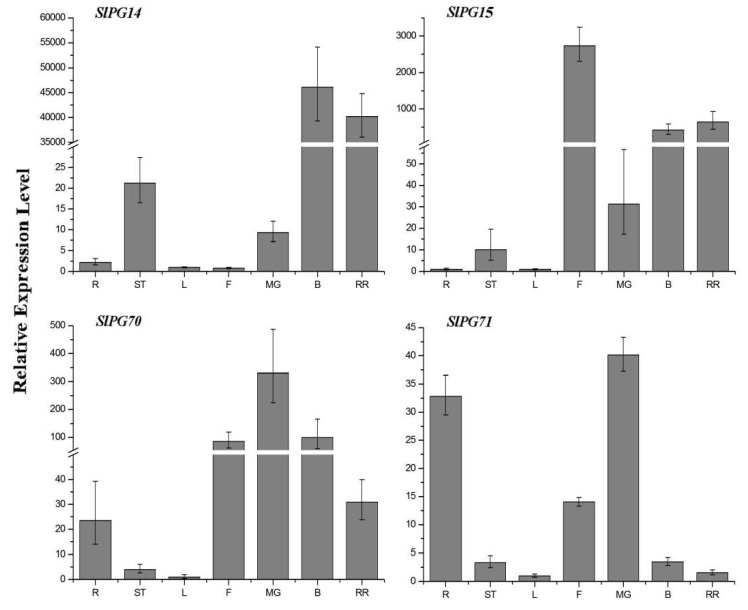
Detailed expression profile analysis of the four SlPG genes based on qRT-PCR analysis in different fruit developmental stages of *S. lycopersicum*. R: roots, ST: stems, L: leaves, F: flowers, MG: mature green fruit, B: breaker fruit, RR: red ripening fruit.

To further analyze the *SlPGs* involved in tomato fruit development, four *SlPGs* (*SlPG14*, *SlPG15*, *SlPG70*, and *SlPG71*) with detectable specific or high expression level in the three stages of fruit developmental process (mature green, breaker, and red ripening fruit) were further analyzed. Meanwhile, it is worth noting that some of the *SlPGs* with relatively low expression levels in fruit developmental processes compared with their expression levels in other tissues were not included in this section, such as *SlPG9*, *SlPG55-1*, and *SlPG58-2* (Figure 4). As shown in Figure 5, the expression level of *SlPG14* in the flowering stage to mature green fruit stage was low. With the fruit development, the *SlPG14* expression increased rapidly along with the fruit development and reached its peak in the fruits of the breaker stage. *SlPG15* showed the highest expression in flower and a relatively high expression in breaker and red ripening fruit. For *SlPG70*, its expression level was low in the early stage of flowering. With the fruit development, its expression peaked at the stage of the mature green stage, and then gradually decreased. Moreover, the expression level of *SlPG71* also peaked at the mature green stage, and then gradually decreased with the fruit development. In addition, the *SlPG71* also had a relatively high expression level in the roots.

The available RNA-seq-based datasets about tomato in TEB and Tomato Expression Atlas (TEA) [33,34] were utilized for further confirming and screening of the main SlPGs participated in the fruit developmental process. The results showed that all of the four SlPGs mentioned above were also found to have a relatively high expression level during the fruit development of another two tomato varieties (“pimp” and “M82”) (Figures S3 and S4). In particular, we found another member of tomato PG gene family (*SlPG49*) also showed a high expression level in the pericarp, indicating that it may play a great role during the fruit ripening process of *S. lycopersicum* L. cv M82. In addition, using the excellent comprehensive tomato fruit transcriptome atlas in TEA, we further investigated the expression profiles of the five fruit-related *SlPGs* along spatial and developmental gradients of tomato fruit development. The results were in consist with our findings that *SlPG14*, *SlPG15*, and *SlPG49* expressed at very high levels in the late stages of tomato fruit development while *SlPG70* and *SlPG71* had specifically higher expressions in early fruit development (Figures S5 and S6). The *SlPG14* and *SlPG49* transcripts were the highest in the pericarp, while *SlPG15*, *SlPG70*, and *SlPG71* in internal fruit tissues such as septum and locular tissue.

### 2.6. Promoter Analysis of the SlPGs

PGs have been suggested to participate in many stages of plant development, such as fruit ripening, organ abscission, pod and anther dehiscence, and pollen maturation [2,3]. Thus, identification and analysis of the regulatory motifs present in the promoters of SlPG genes are beneficial in expanding the current information on the molecular regulation during tomato developmental processes mediated by numerous transcription factors and various plant hormones. The *cis*-elements related to plant hormone response, environmental stress response, and specific organ/tissue development in their promoter sequences were assayed using PlantCARE [35] to understand the transcriptional regulation and their potential functions. Eleven types of *cis*-elements which responded to plant hormones, eight types of *cis*-elements related to environmental stress, and eight types of *cis*-elements related to specific organ/tissue development were presented in the 1.5 kb upstream sequences of the 54 *SlPGs* representing their promoter regions (Table S4). All members of these *cis*-elements in each promoter regions of the 54 *SlPGs* were shown in Supplemental Figure S7 and Table S5. All of the SlPG promoters contained more than three *cis*-elements, of which the promoter of *SlPG20-2* contains 18, the most *cis*-elements. Each promoter of *SlPGs* included at least two of the three main types of *cis*-elements except for *SlPG38-1*, the promoter of which only contained environmental stress-associated *cis*-elements. In particular, the endosperm expression related *cis*-regulatory element exhibited in the promoter regions of three fruit development related SlPG genes (*SlPG14*, *SlPG15,* and *SlPG71*). The *cis*-acting element involved in salicylic acid responsiveness was detected within the upstream promoter regions of four out of the five fruit development related *SlPGs* (except for *SlPG15*). However, we failed to detect a direct link between the composition of the *cis*-elements and their main expression patterns in the different Groups divided according to the tissue specificity and relative gene expression levels. 

## 3. Discussion

PG is a type of hydrolases involved in the modification of pectin networks in plant cell walls. In recent years, genome-wide data on many important vegetable crops have been announced [36,37], which provides unprecedented convenience and opportunity to investigate the commonness, characteristics, and evolution of all members of a gene family from the whole genome level. However, to our knowledge, only a small number of tomato PG genes were cloned (Figure 3, Table 1).

In the present study, based on the analysis of the PG gene family, the nucleotide sequence of the target region in the genome was re-predicted, and the conserved domain was analyzed in *S. lycopersicum*. Finally, 54 PG gene family members of tomato were determined, which were renamed according to the homology of *S. lycopersicum* and *A. thaliana*. It has been reported that most PG members from different species contain four conserved domains [9]. Domains I and II may constitute catalytic sites, domain III may participate in the reaction, and domain IV constitutes a possible candidate for the interaction with the ionic groups of the carboxylic acid groups in the substrate [9]. Domain III shows lower conservation and is missing in PGs belonging to clade E, and a PG gene can be identified as containing at least one of the four conserved domains [7,8,9,29]. Of the 54 PGs, 40 contained four conserved domains (Figure 1, Table 1). In accordance with the previous reports, the 12 SlPGs that lacked the third conserved domain also belonged to the clade E (Figure 1 and Figure 3; Table 1), indicating that members of this clade were also conserved in *S. lycopersicum* in the process of evolution and may have a unique role in specific organ/tissue development. One member that belonged to clade F (*SlPG70*) did not have the first three domains that are important for its catalytic reaction, indicating that it may be a new type of PG that plays a unique role in development process of *S. lycopersicum* or a pseudogene that have lost the catalytic ability of a PG during the evolutionary process. Previous studies showed that the encoded protein QRT3 in *A. thaliana* lacking the four conserved domains also had homogalacturonan hydrolase activity [8,30]. Therefore, the homolog of *AtQRT3* in *S. lycopersicum* (*SlPG71*) was still considered to be one of the *SlPGs* though it contained none of the four domains. Moreover, chromosome localization analysis of the tomato PG genes showed that they were not randomly distributed on 12 chromosomes, and 10 clusters of SlPG genes presented in the genome of *S. lycopersicum* (Figure 2, Table 1). There were three clusters of tandem-duplicated genes present in Ch12, and one each in Ch02 and Ch03, indicating that tandem duplication may be one of the factors responsible for the expansion of ancestral *SlPGs* after the divergence of *Arabidopsis*-*S. lycopersicum* in the evolutionary process of tomato. By comparing the homologous genes of them in other Solanaceae species, we found that three tandem duplications (*SlPG21-1*/*SlPG21-2*, *SlPG24-2*/*SlPG24-7*, and *SlPG24-5*/*SlPG24-8*/*SlPG24-9*) happened after the divergence of tomato from pepper but before the divergence of tomato from potato, while the other two (*SlPG24-1*/*SlPG24-4* and *SlPG56-2*/*SlPG56-3*) happened before the divergence of tomato from pepper. In particular, four of the five clusters of tandem-duplicated SlPG genes came from the Clade C indicating that the special expansion of PG gene family in the evolutionary process of *S. lycopersicum* maybe essential for serving the species-specific traits or organ/tissue development.

As one of the largest gene family of plants, the PG gene family has experienced complex evolutionary events in the long evolutionary process of plants. Phylogenetic analysis of the PG gene family has been carried out in many studies, from the initial classification of three Clades (A, B, and C) to the adjustment and expansion of the six and seven Clades (A to F and A to G) [2,5,10,12]. In this study, the tomato PG gene family was classified by the A to G classification according to the analysis of their phylogenetic tree as in the previous research about the PG gene family of *C. sativus* and *B. rapa* [8,12]. The analysis of the structure of introns and exons and the conserved motif and domain of tomato PG genes revealed that Clades A, B, and F contained relatively more introns. In addittion, the structures of the introns and exons were relatively conserved among the members of the clade, as compared to the other clades. However, there was a significant difference between the members of different clades. This suggested that there were significant differences in the number and distribution of genetic structures and conserved motifs among members of different subfamilies, but the subfamily members were very similar and conserved. These results further illustrate that different members of the PG gene family have differentiated in the gene structure and conserved motifs during the long evolution of plant and have formed a number of subfamilies with different sequence structural features. The differences in gene structure and conserved motif between different subclass of PG genes may be related to their different functions in plant development.

Many studies have reported that transcript abundance in particular organs at a given time is an important prerequisite to the functional elucidation of the corresponding genes required for the specific developmental processes [8]. In this study, we investigated the expression profiles of the 54 SlPG genes in roots, leaves, stems, flowers, and fruits of three different development stages (Figure 4). The SlPG genes could be clustered into six groups according to the expression patterns in different tomato tissues based on the hierarchical clustering results. In *B. rapa* and *C. sativus*, most BrPG and CsPG genes in Clade E were found to be ubiquitously expressed in different tissues [8,12]. Expression patterns of PG genes in the other two dicots (*Glycine max* and *Medicago truncatula*) and two grasses (*Zea mays* and *O. sativa*) also showed that most of the clade E members could be detected at high overall expression levels in all tissues [7]. In accordance with previous research, we found that more than half *SlPGs* of Group I belonged to Clade E and were also defined as being expressed ubiquitously (Figure 4, Table S3) which further proved the previous theory that the Clade E members of PG family are possibly ancient proteins and are fundamental and indispensable in almost all plant organs of different species [7,10]. The PG genes of Clades C, D, and F may be associated with flower development, and the main expression pattern of PGs in clades C and F may be different between the grasses and dicots [7,9,10,12]. Coincidentally, most PGs of Clades C and D can also be observed to show flower-specific expressions in tomato. However, Clade F, on the one hand, was apparently different and the four members of it belonged to three Groups showing diverse expression patterns. A previous study demonstrated that the expression patterns of the members of Clades A, B were not so conserved across species, and members of the two clades showed divergent expression profiles among different species [7]. Similar situations occurred in both SlPGs of clades A and B; that is, members of those tomato PGs were all clustered into more than three Groups and were observed to have a divergent expression profile. Therefore, it also further contradicts the conclusion that the PGs of clades A and B are mainly related to fruit and abscission zone development as discussed in one of our previous researches [2,7,9]. In addition, our findings of the expression patterns of SlPG genes were confirmed by investigating the available RNA-seq-based datasets in TEB, and most of the SlPG genes were found to have a similar expression profile among different tomato organs (Figure S2). Therefore, our findings would be of great help for further elucidating the precise biological function of SlPGs in the development of different organs or tissues. 

PG plays an important role in the complex physiological and biochemical process of fruit softening associated with extensive pectin disassembly, which may increase the pore size of the pectin network, resulting in cell wall swelling [2]. It was interesting to find that only five members of the tomato PG gene family (*SlPG14*, *SlPG15*, *SlPG49*, *SlPG70*, and *SlPG71*) were specifically or highly expressed in the fruits of three different development stages (Figure 4, Figure 5, and Figure S3–S6). Phylogenetic analysis showed that *SlPG14* and *SlPG15* belonged to the B clade, *SlPG49* belonged to clade E, and the other two belonged to the F and G clades, respectively (Figure 3). Specifically, *SlPG14* is exactly the tomato PG gene *pTOM6* that has been previously studied through the comparison analysis of the coding sequences of them [22,38]. As shown in the previous study, suppression of that gene by 99% cannot reduce pectin solubilization and alter the fruit softening [24,39]. However, that suppression can repress the pectin depolymerization and change the storage life of overripe fruits, postharvest pathogen susceptibility, and viscosity of processed tomato paste [24,40]. Therefore, those studies led to the hypothesis that PG activity alone may be insufficient to affect texture, but it contributes significantly to tissue deterioration in the later stages of ripening [2,41]. However, PGs are encoded by a typical large gene family in higher plants [29], and it is impossible to identify all SlPG genes that are associated with the development of tomato fruit while the genome sequence of tomato was not released. It has been reported that at least three MaPG genes may be responsible for softening in banana during ripening [15]. Two *FaPGs* are highly expressed during the ripening process of strawberry, and only the silencing of the *FaPG1* significantly reduces the softening of ripened fruits at harvest and after several days of storage [26]. Moreover, 16 *CsPGs* are evidently expressed in five different stages of the developmental process of fruit in *C. sativus*, which can be divided into three groups [8]. Therefore, the functional redundancy of different members of PG gene family must exhibit in the softening or ripening process of fruit development. In the present study, except for *SlPG14* whose function has already been characterized in previous studies, another four *SlPGs* (*SlPG15*, *SlPG49*, *SlPG70*, and *SlPG71*) were also found to be involved in the development of tomato fruit although *SlPG70* and *SlPG71* were mainly expressed in the mature green stage of fruit (Figure 4, Figure 5, and Figure S3–S6). *SlPG15* was reported to be the first functional sterility gene (*ps-2*) isolated in the Solanaceae family and was proved to have a relatively high expression level in maturing fruits, suggesting that it might play an important role in fruit maturation [42]. However, there are no available results about the exact role of *ps-2* in the fruit ripening process of tomato to date, meaning that the underlying mechanism of cell wall remodeling in tomato remains need more efforts to provide new insights. Further elucidation of the biological function of *SlPG15*, *SlPG49*, *SlPG70*, and *SlPG71* is of great importance for revealing the molecular mechanism that controls the fruit maturation and for improving the quality and yield of tomato. 

It is also interesting to note that the homologous genes of *SlPG15* and *SlPG71* in Arabidopsis are exactly the *AtQRT2* and *AtQRT3* respectively, which have been proved to participate in pectin degradation in the pollen mother cell wall during the tetrad stage of pollen development [4,30]. Knockout of *AtQRT2* and *AtQRT3* leads to the generation of tetrad pollen because of the failure to degrade pectin in the pollen mother cell wall during the tetrad stage [4,30]. In our previous research, one of the two homologous genes of *AtQRT3* in *C. sativus* (*CsQRT3-2*) was also found to be highly expressed in fruits at 27 days after pollination, while the other one *CsQRT3-1* was highly expressed in the female flower [8]. Therefore, it could be concluded that the two PGs, QRT2 and QRT3, play different roles in the process of development among different species after a long evolutionary process. 

Furthermore, the *cis*-elements for plant hormone response, environmental stress response, and specific organ/tissue development in the promoter sequences of the 54 SlPG genes were also investigated in this study. At least three *cis*-elements belonging to the three major categories were found to exhibit in the promoter of each SlPG, indicating that the expression and function of them were precisely regulated by plant hormones, environmental stress, and other regulatory factors. That is consistent with the role of PGs in various plant developmental processes that need precise regulations, such as fruit ripening, organ abscission, etc. The endosperm expression related *cis*-regulatory element was found in the promoter regions of three fruit development related SlPG genes (*SlPG14*, *SlPG15*, and *SlPG71*), which further supported their participation in the *S. lycopersicum* fruit development. Salicylic acid is an important plant hormone regulating both local disease resistance mechanisms and systemic acquired resistance in plants [43]. We found that the promoters of four out of the five fruit development related SlPGs contained the *cis*-acting element involved in salicylic acid responsiveness, implying that the corresponding hormone may have an important role in regulating the fruit developmental process of tomato or those fruit-related SlPGs may also participate in the disease resistance process regulated by salicylic acid during the tomato fruit development and ripening. However, we failed to detect the ethylene-responsive element (ERE), which plays an important role in the regulation of many genes related to fruit ripening [44], within the upstream promoter regions of the five fruit development related SlPGs, suggesting that other unproven *cis*-elements related to fruit-specific regulation may be present in their promoter regions. Additionally, we were unable to detect a direct link between the composition of the *cis*-elements and their main expression patterns of the six main groups, further implying that the regulation of PG family members is very complex and implicated with multiple regulatory factors. 

In conclusion, a comprehensive analysis was conducted on whole-genome annotation, genomic structures, and molecular evolution of PG genes in *S. lycopersicum*. The expression profiles and the upstream regulatory *cis*-elements of the SlPG genes were analyzed. Our results demonstrated that tomato PG genes were not randomly distributed on the twelve chromosomes and could be classified into seven clades, and within each clade the exon/intron structures were conserved. qRT-PCR results and RNA-seq-based datasets analysis indicated that most SlPGs had specific or high expression patterns in at least one organ, and five PG genes were detected to participate in the tomato fruit development process. Promoter analysis showed that more than three *cis*-elements associated with plant hormone response, environmental stress response or specific organ/tissue development exhibited in each SlPG promoter region. The final challenge is to define the specific functions of the rest SlPG genes that have not been clearly characterized during plant development and in response to environmental factors as well as plant hormones. In addition, the precise mechanism of fruit softening has been the subject of decades of research but remains elusive. We have established a highly efficient transformation system in tomato, and we are optimistic to focus on revealing the functions of the fruit development related PGs and obtaining ripening-controlled crops via genetic improvement.

## 4. Materials and Methods

### 4.1. Identification of Tomato PG Family Genes

On the basis of the amino acid sequence of the 68 Arabidopsis PG genes identified and systematically nominated in a previous research [12], TBLASTN searches were conducted in tomato genome database. In detail, TBLASTN search of Arabidopsis PG protein sequences were carried out in the tomato genome database v2.5 (available online: http://solgenomics.net/) with default algorithm parameters. Then, each amino acid sequence of the representative PGs of glycosyl hydrolase family 28 in Pfam database (PF00295) (available online: http://pfam.xfam.org/family/PF00295) was used as a query in the TBLASTN search for potential PG gene family sequences in the tomato database. Finally, combining the results above, all of the candidates in the tomato genome, together with flank regions of 5 kp upstream and downstream of each candidate, were re-annotated by using FGENESH (available online: http://linux1.softberry.com/berry.phtml?topic=fgenesh&group=programs&subgroup=gfind). The putative proteins that contained more than one highly conserved domains (domain I, II, III, and IV) of PGs [9] were regarded as PGs. Additionally, the PGs encoded by the homologous genes of the well-known PG *AtQRT3* were also analyzed [30]. The molecular weights and isoelectric points of the deduced tomato PG proteins were predicted using the Compute pI/Mw tool of ExPASy (available online: https://web.expasy.org/compute_pi/), and their signal peptide sequences and subcellular localization were analyzed using SignalP 4.1 (available online: www.cbs.dtu.dk/services/SignalP/) [45] and TargetP 1.1 (available online: http://www.cbs.dtu.dk/services/TargetP/) [46].

### 4.2. Chromosome Localization and Sequence Characterization

The whole sequences of tomato chromosome were downloaded from the tomato genome database v2.5 (available online: http://solgenomics.net/). The SlPG sequences were used as query sequences to detect the precise locations of genes on *S. lycopersicum* chromosomes using Oligo 6.0 (Molecular Biology Insights, Colorado Springs, CO, USA). The locations of all *SlPGs* were determined. Tandem-duplicated genes and segment-duplicated events were analyzed according to a previous study [47]. CoGe comparative genomics system [31] and Circos [32] were used to conduct the synteny analysis and generate the diagram of genomic distribution and syntenic relationship of *SlPGs*.

### 4.3. Intron-Exon Structure and Phylogenetic Analyses

The structure of the intron and exon of the SlPG genes were analyzed using GSDS (available online: http://gsds.cbi.pku.edu.cn/index.php). Phylogenetic analysis was performed by the method of BI using MrBayes 3.1.2 (University of California, San Diego, CA, USA) [48]. Posterior probabilities (PPs) were calculated under the general time-reversible (GTR) model [49,50], assuming that rate variation across sites followed a discrete gamma distribution with four rate categories [51]. Default priors in MrBayes 3.1.2 were used. Markov chain Monte Carlo (MCMC) data simulation was used to estimate the posterior probability. The Markov chain was allowed to run for 1 million generations, taking a random tree as a starting tree and samples every 1000 generations. The first 250 burn-in samples were discarded from the obtained samples, and the common tree was obtained. The reliability of the BI topology tree was viewed by the TreeView program (available online: https://www.treeview.co.uk/). 

### 4.4. Plant Materials and Treatment

Tomato plants (*S. lycopersicum* L. cv Micro-Tom) were purchased at the Tomato Genetic Resources Center (University of California, Davis, CA, USA). The tomato plants were grown in the 600 mL pots (one seedling per pot) containing a mixture of peat: vermiculite (1:1) and cultured in an artificial climate chamber with day/night temperatures 26/20 °C, and 16/8 h light/dark (60–70% relative humidity) in Zhejiang A&F University, Hangzhou, China. The roots, flowers, stems, leaves, and fruits (mature green, breaker, and red ripening stage) [52] were collected during the fruit development period. All the materials were mixed and frozen in liquid nitrogen immediately and stored at −80 °C.

### 4.5. Expression Analysis of SlPG Genes by qRT-PCR

Total RNA was extracted using MiniBEST Plant RNA Extraction Kit (Takara, Japan). The first strand cDNA was prepared using Primer Script RT reagent kit (Takara, Japan). The expression patterns of tomato PG gene family in different tissues and organs and fruits with different development stages were analyzed using qRT-PCR. Gene-specific primers were designed based on the result of the multiple-alignment of SlPG gene sequences using Primer 5.0 (PREMIER Biosoft International, Palo Alto, CA, USA) (Table S6). The gene-specific qRT-PCR primers were designed according to the non-conserved region of gene coding sequence. Through multi-sequence alignment, specific primers were designed for the PG gene family members of tomato. The specificity of each primer pair to its corresponding gene was verified through the BLASTN program in the tomato genomic database. Part of the PG genes had a high sequence similarity, so similar tomato PG genes were classified into a gene to design its universal primers for expression analysis. The tomato inner control gene Ubi3 [53] was used as the internal reference gene (Table S6). QRT-PCR analysis was performed using Takara’s SYBR Premix Ex Taq kit on a ABI 7300 machine (Thermo, Waltham, MA, USA). The amplification program was 95 °C for 30 s, and 40 cycles of 95 °C for 5 s and 54 °C for 30 s. Data were normalized to the expression level of the inner control genes. Two biological and three technical replicates for each sample were performed. The quantitative data was processed with the 2^−ΔΔ*C*t^ method [54], and the heatmap was generated by Cluster 3.0 (Stanford University, Stanford, CA, USA) [55]. To compare the expression patterns of SlPGs, we acquired their RNA-seq-based data from the TEB (available online: http://bar.utoronto.ca/efp_tomato/cgi-bin/efpWeb.cgi) and TEA (available online: http://tea.solgenomics.net/expre ssion_viewer/input) [33,34].

### 4.6. Cis-Regulatory Elements Analysis of SlPG Genes

To investigate the *cis*-elements in the promoter sequences, 1.5 kb of genomic DNA sequences upstream of the initiation codon (ATG) of each *SlPG* were obtained from the tomato database. The elements in the promoter sequences were analyzed through the PlantCARE [35] (available online: http://bioinformatics.psb.ugent.be/webtools/plantcare/html/).

## Figures and Tables

**Table 1:** The polygalacturonase (PG) gene family in *Solanum lycopersicum.*

Gene *	*Solanum lycopersicum* Locus	Chr	Location (5′–3′)	Predicted Protein (aa)	Molecular Weight (kDa)	Isoelectric Points (pI)	Signal Peptides (aa)	Sub ** Cellular Locationaa	Domains
*SlPG38-1*	Solyc00g030510.2.1	0	12605208–12607452	403	42.47	6.96	1–23	S	I II III IV
*SlPG37-4*	Solyc01g009640.1.1	1	3883446–3879488	391	42	6.81	1–22	S	I II III IV
*SlPG44*	Solyc01g066070.2.1	1	65439007–65436858	405	43.51	7.92	1–25	S	I II III IV
*SlPG4*	Solyc01g079130.1.1	1	70719258–70721952	472	51.51	5.96	1–30	S	I II III IV
*SlPG16*	Solyc01g087280.1.1	1	73976402–73980711	425	47.65	9.46	1–22	S	I II III IV
*SlPG55-2*	Solyc01g094970.2.1	1	78127060–78124570	475	51.83	6.63	1–24	S	I II IV
*SlPG7*	Solyc01g100980.2.1	1	82616598–82614439	476	52.16	7.44		S	I II III IV
***SlPG24-8 (TAPG1)***	Solyc02g067630.2.1	2	32341911–32343450	392	41.85	8.87	1–17	S	I II III IV
***SlPG24-9 (TAPG2)***	Solyc02g067640.2.1	2	32347519–32349041	392	41.87	8.49	1–17	S	I II III IV
***SlPG24-5 (TAPG3)***	Solyc02g067650.1.1	2	32355651–32357075	389	41.75	8.34	1–17	S	I II III IV
*SlPG71*	Solyc02g068400.2.1	2	32931014–32932787	481	51.66	5.92	1–22	S	
*SlPG43*	Solyc02g069480.1.1	2	33931720–33929059	393	42.08	6.4	1–28	S	I II III IV
*SlPG56-2*	Solyc03g007940.2.1	3	2435889–2432960	467	50.81	5.67	1–31	S	I II IV
*SlPG56-3*	Solyc03g007950.2.1	3	2441660–2439216	467	50.95	6	1–31	S	I II IV
*SlPG69*	Solyc03g113230.1.1	3	57495929–57493305	394	43.12	5.72	1–22	S	I II III IV
***SlPG17-1 (XPG1)***	Solyc03g116500.2.1	3	59953586–59957497	452	49.44	8.83		S	I II III IV
*SlPG68*	Solyc03g116580.2.1	3	59990295–59986839	437	47.05	8.11	1–24	S	I II III IV
*SlPG57*	Solyc03g117750.2.1	3	60847079–60843135	487	54.84	9.26		M	I II IV
*SlPG6*	Solyc04g008230.2.1	4	1904669–1901842	490	52.87	4.88	1–24	C	I II III IV
***SlPG15 (PS-2)***	Solyc04g015530.1.1	4	5755310–5764191	463	50.99	7.49			I II III IV
*SlPG12-2*	Solyc04g025440.2.1	4	20488981–20492100	460	49.82	6.33	1–18	S	I II III IV
*SlPG11*	Solyc05g005040.2.1	5	62026–58076	471	52.07	9.04	1–28	S	I II III IV
*SlPG9*	Solyc05g005170.2.1	5	159216–162264	508	55.67	8.7	1–30	S	I II III IV
*SlPG55-1*	Solyc05g049980.2.1	5	58973161–58969921	486	53.4	5.57		S	I II IV
*SlPG45*	Solyc06g009200.2.1	6	3145482–3148100	410	43.42	6.78	1–31	S	I II III IV
*SlPG48-1*	Solyc06g009790.2.1	6	3813956–3809878	457	49.35	5.09	1–22	S	I II IV
*SlPG52*	Solyc06g060170.2.1	6	34525051–34519529	488	53.94	8.63			I II IV
*SlPG17-2*	Solyc06g068040.2.1	6	38555860–38558728	437	48.58	9.19		S	I II III IV
*SlPG8*	Solyc07g015870.2.1	7	5634411–5629702	449	49.21	5.9	1–26	S	I II III IV
*SlPG37-2*	Solyc07g041650.1.1	7	51162465–51164213	389	41.82	7.53	1–22	S	I II III IV
*SlPG58-2*	Solyc07g042160.2.1	7	52482303–52486747	493	55.05	9.3			I II IV
*SlPG38-2*	Solyc07g044870.2.1	7	55248946–55251510	405	43.3	7	1–23	S	I II III IV
*SlPG37-1*	Solyc07g056290.1.1	7	61470406–61473987	401	43.96	8.54		S	I II III IV
*SlPG20-1*	Solyc08g014540.1.1	8	4615191–4612405	395	42.92	9.2	1–25	S	I II III IV
*SlPG21-3*	Solyc08g014560.1.1	8	4650562–4654908	396	43.39	9.47		S	I II III IV
***SlPG64 (PGcat)***	Solyc08g060970.2.1	8	43357711–43354556	423	46.31	8.2		S	I II III IV
*SlPG56-1*	Solyc08g081480.2.1	8	61693642–61696788	483	52.9	5.18		S	I II IV
*SlPG49*	Solyc08g082170.2.1	8	62200995–62196897	467	50.97	8.35	1–25	S	I II IV
*SlPG48-2*	Solyc09g075460.2.1	9	62665686–62668281	446	48.62	5.3	1–28	S	I II IV
*SlPG12-1*	Solyc09g098270.2.1	9	67421613–67424507	433	47.3	4.95			I II III IV
*SlPG36*	Solyc10g047570.1.1	10	36889700–36892063	392	41.9	6.29	1–22	S	I II III IV
*SlPG37-3*	Solyc10g047590.1.1	10	36929234–36932108	387	42.31	9.01	1–22	S	I II III IV
***SlPG14 (pTOM6)***	Solyc10g080210.1.1	10	60890335–60883700	457	50.05	6.4	1–24	S	I II III IV
*SlPG58-1*	Solyc12g009210.1.1	12	2490735–2496842	495	55.53	8.98		M	I II IV
*SlPG70*	Solyc12g009420.1.1	12	2702056–2697739	426	46.34	7.47		S	IV
*SlPG20-2*	Solyc12g019120.1.1	12	9156572–9152746	395	43.08	8.9	1–27	S	I II III IV
*SlPG21-2*	Solyc12g019130.1.1	12	9245985–9244294	390	42.5	9.66	1–24	S	I II III IV
*SlPG21-1*	Solyc12g019140.1.1	12	9293132–9295123	389	42.4	9.77	1–23	S	I II III IV
***SlPG24-6 (TPG7)***	Solyc12g019180.1.1	12	9461467–9459617	397	42.15	6.05	1–25	S	I II III IV
*SlPG24-2*	Solyc12g019220.1.1	12	9700427–9702341	395	42.09	9.08	1–25	S	I II III IV
*SlPG24-7*	Solyc12g019230.1.1	12	9708477–9710424	391	42.59	8.7	1–24	S	I II III IV
***SlPG24-3 (TAPG6)***	Solyc12g096730.1.1	12	63866593–63868349	395	42.42	8.34		S	I II III IV
***SlPG24-1 (TAPG5)***	Solyc12g096740.1.1	12	63870294–63868858	387	41.31	8.5	1–17	S	I II III IV
***SlPG24-4 (TAPG4)***	Solyc12g096750.1.1	12	63877420–63878834	387	41.37	6.44	1–17	S	I II III IV

* The blod font illustrates the SlPGs that have been reported by previous studies. Text in parentheses indicates the names of the SlPGs used in previous research. ** S, secretory pathway; M, mitochondrion; C, chloroplast.

## Supplementary Materials

Supplementary materials can be found at http://www.mdpi.com/1422-0067/19/8/2290/s1.
