# Supplementary material for: Genome-Wide Identification and Analysis of Polygalacturonase Genes in Solanum lycopersicum
Source: Int J Mol Sci. 2018 Aug 4;19(8):2290. doi: 10.3390/ijms19082290 (PMC6121401; doi:10.3390/ijms19082290)
Supplement: Supplementary file 1 [file ijms-19-02290-s001.zip › ijms-335481-suppl.pdf]

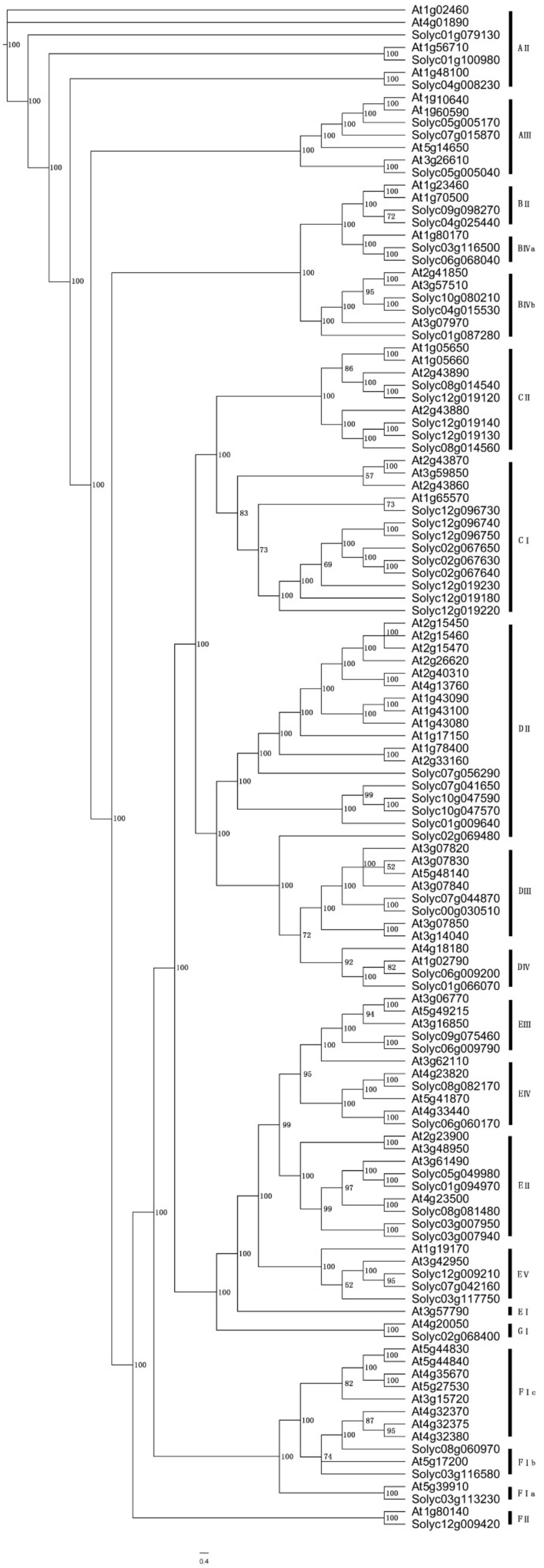

Figure S1. Phylogenetic analysis of PG genes in *Solanum lycopersicum* and *Arabidopsis thaliana*. The *capital letters A* to *G* represent the seven clade numbers.

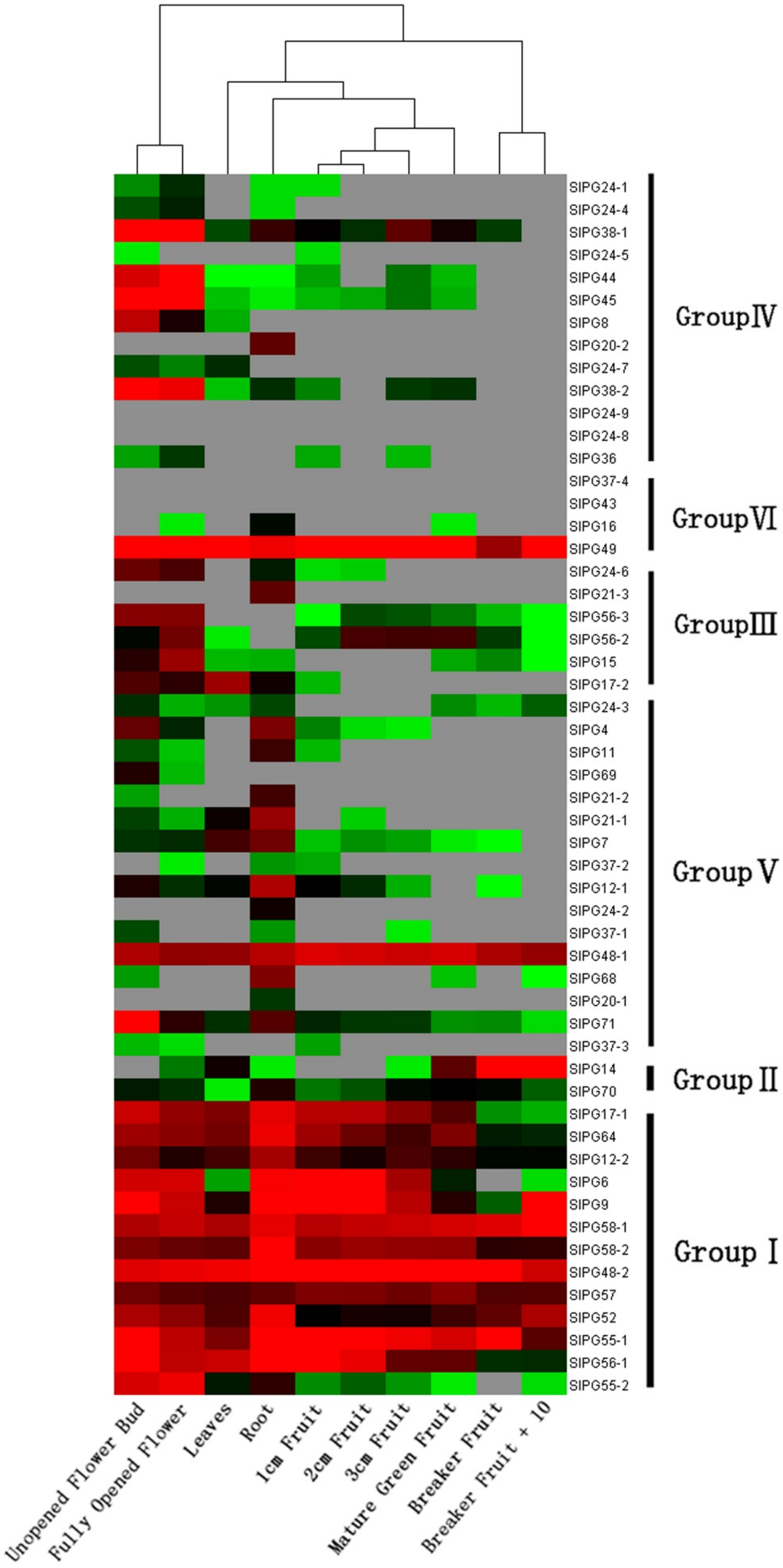

Figure S2. Hierarchical clustering and heat map generated by Cluster 3.0 using the acquired RNA-seq-based data of *SlPGs* from the Tomato eFP Browser, showing their expression levels in the ten different organs/tissues of *S. lycopersicum*.

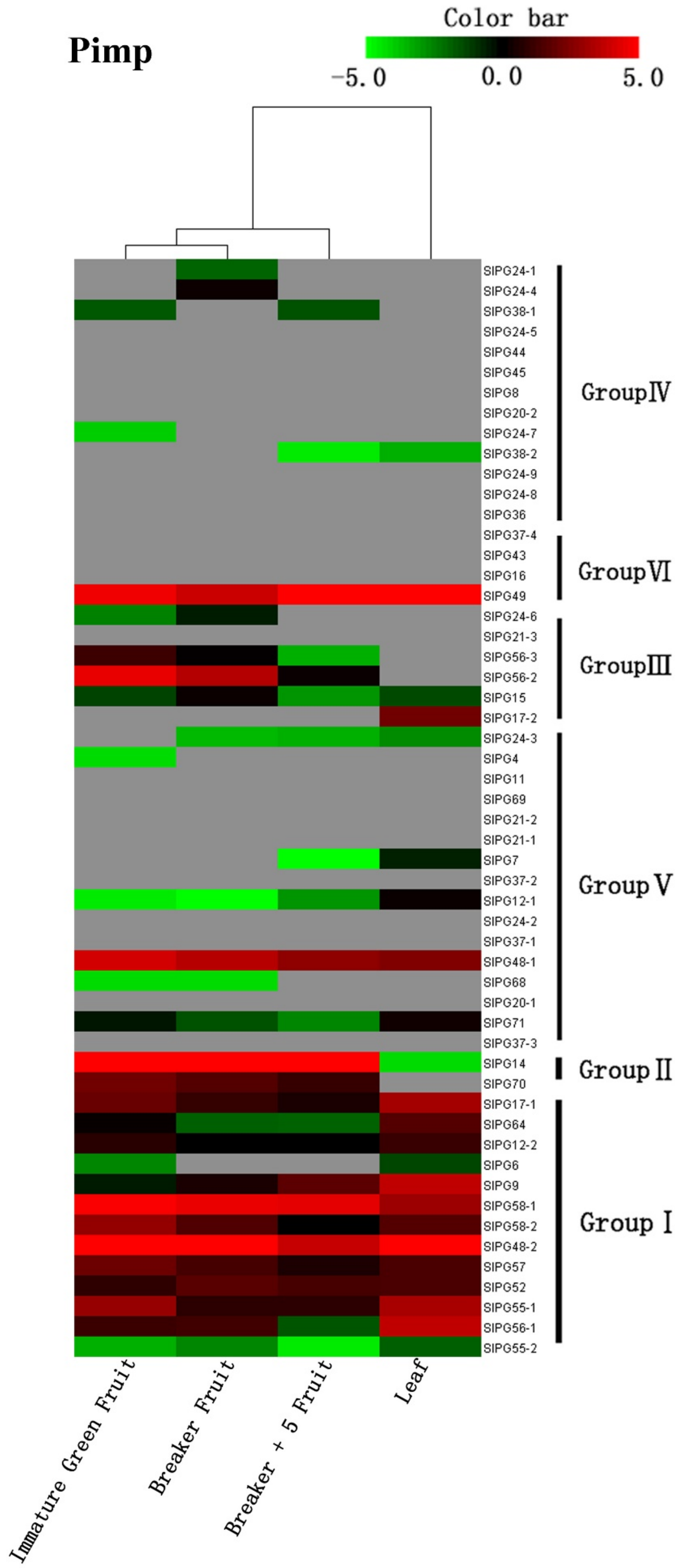

Figure S3. Hierarchical clustering and heat map generated by Cluster 3.0®using the acquired RNA-seq-based data of *SlPGs* from the Tomato eFP Browser, showing their expression levels in the fruit developmental process of *Solanum lycopersicum* L. cv Pimp.

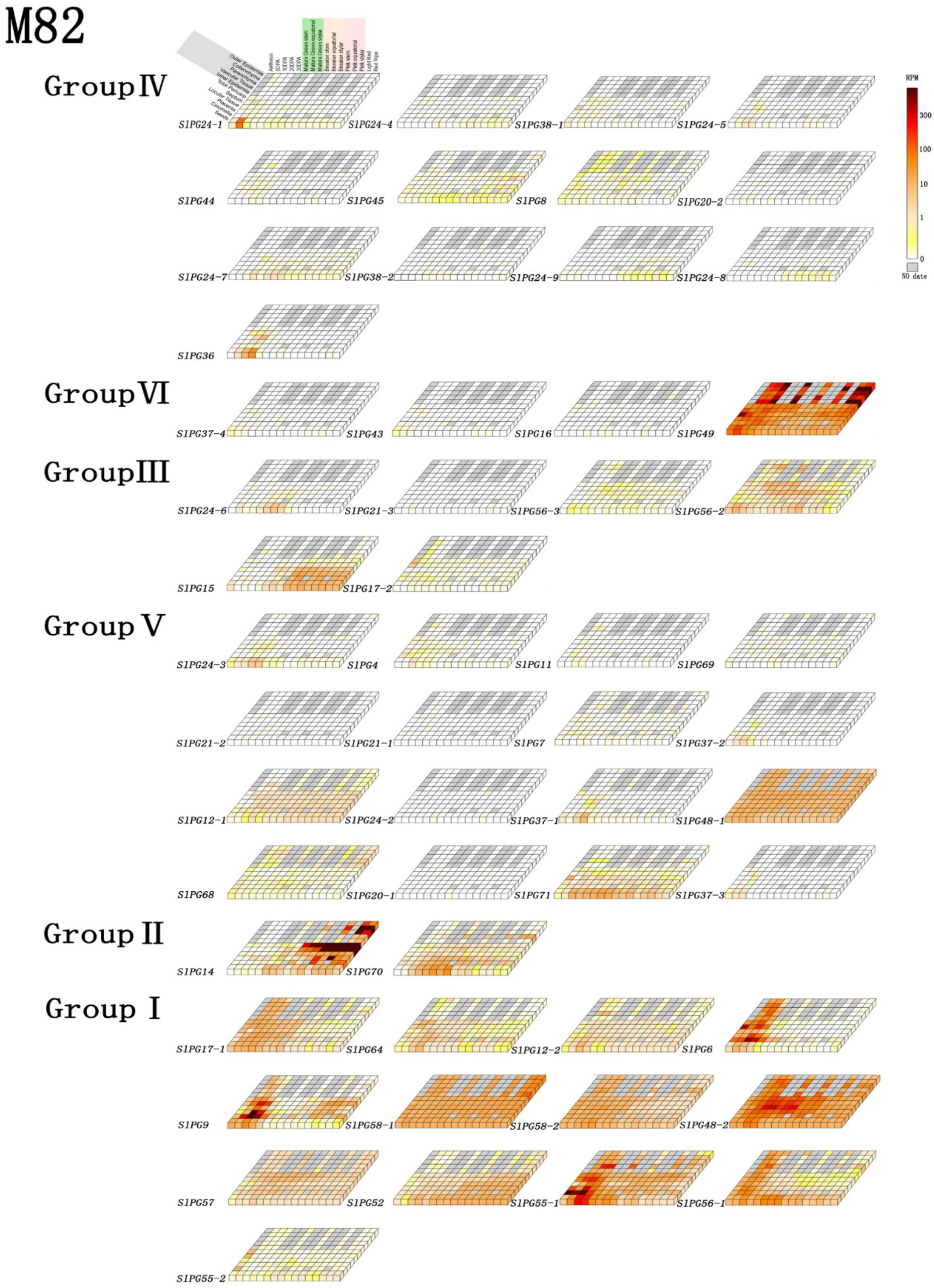

Figure S4. Heat map generated by the online tool of Tomato Expression Atlas showing the expression levels of *SlPGs* along spatial and developmental gradients of tomato fruit development of *S. lycopersicum* L. cv M82.

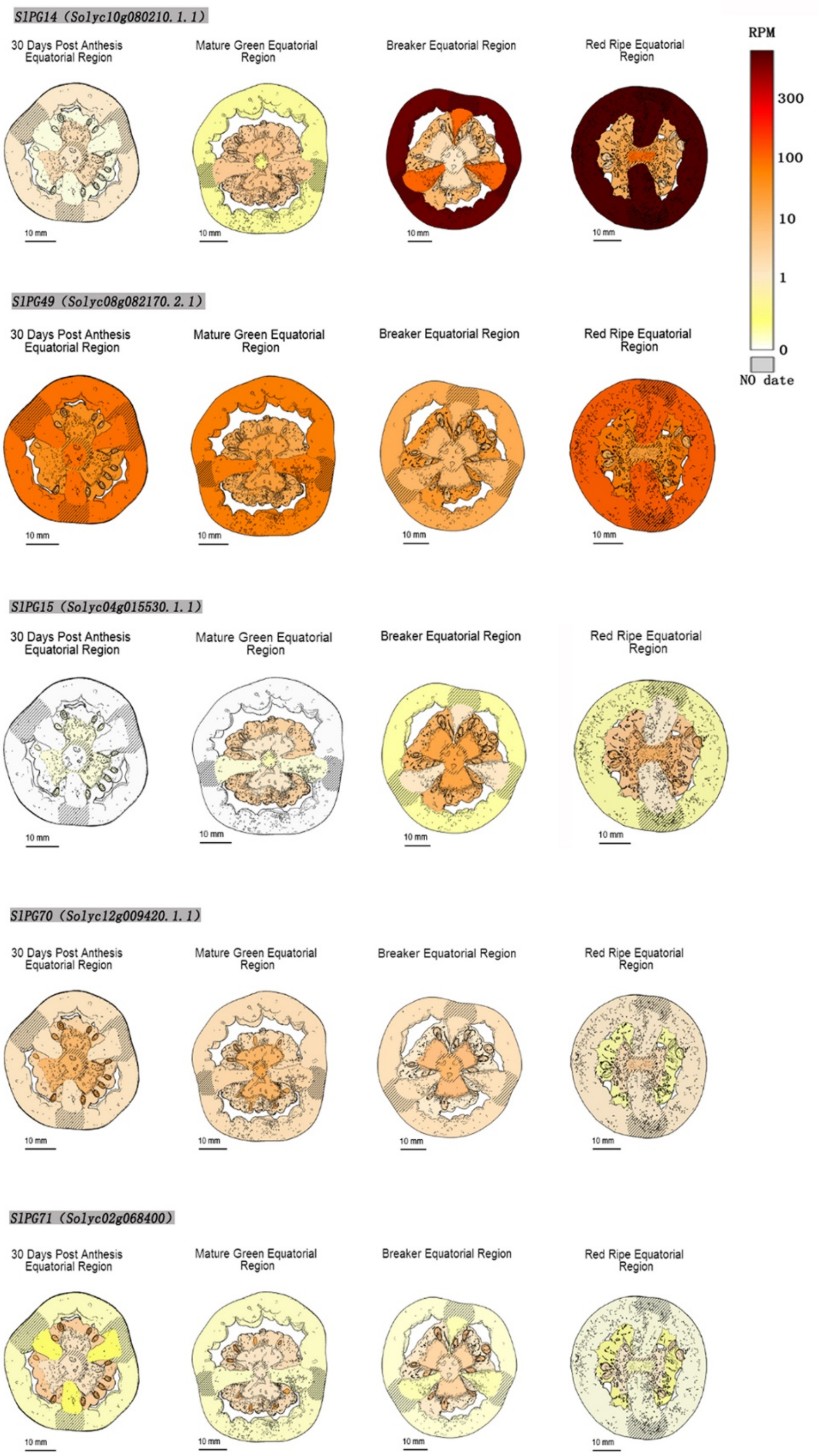

Figure S5. Heat map generated by the online tool of Tomato Expression Atlas showing the expression levels of the five fruit development related *SlPGs* in equatorial region of the four stages of tomato fruit development.

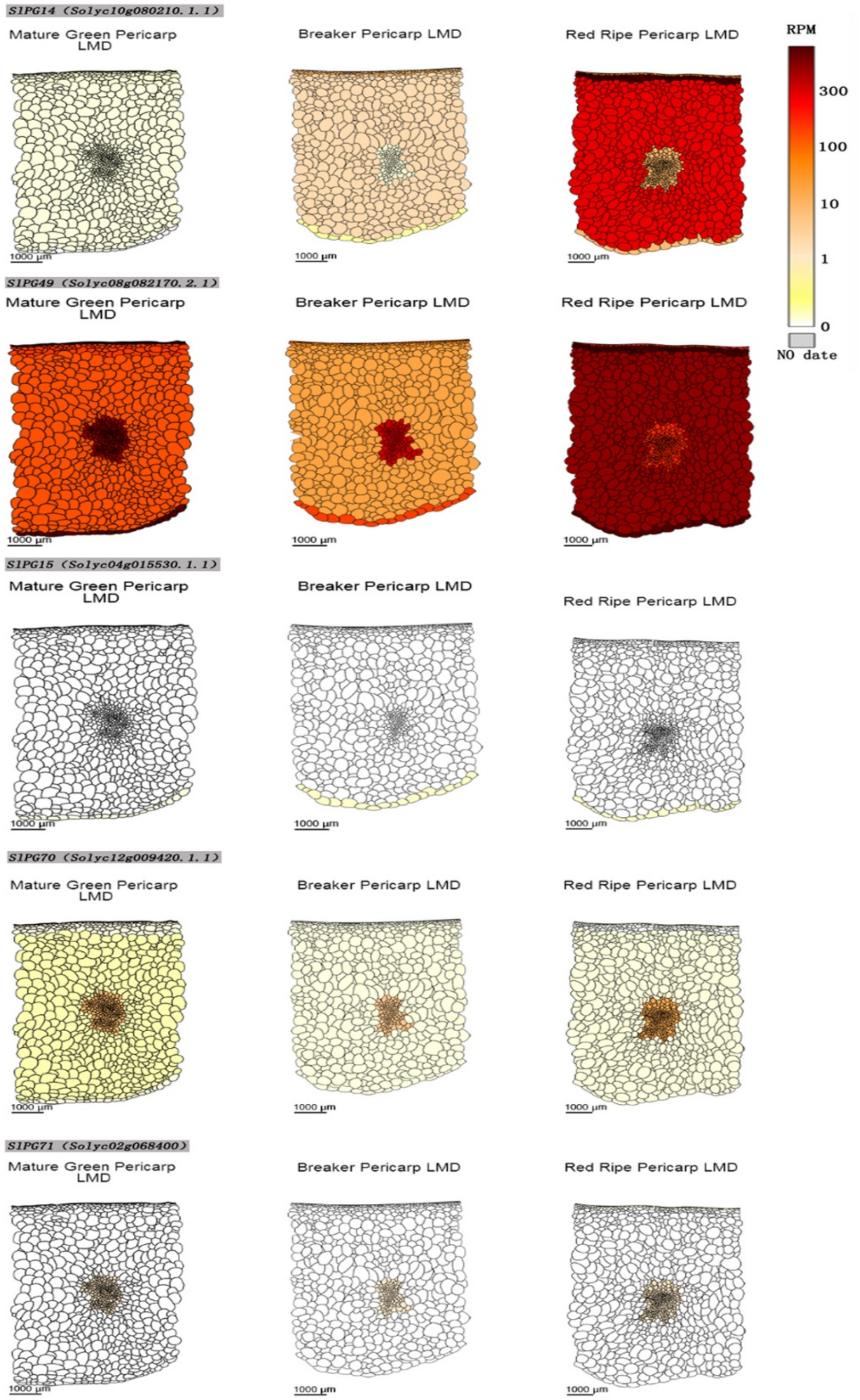

Figure S6. Heat map generated by the online tool of Tomato Expression Atlas showing the expression levels of the five fruit development related *SlPGs* in pericarp of the three stages of tomato fruit development.

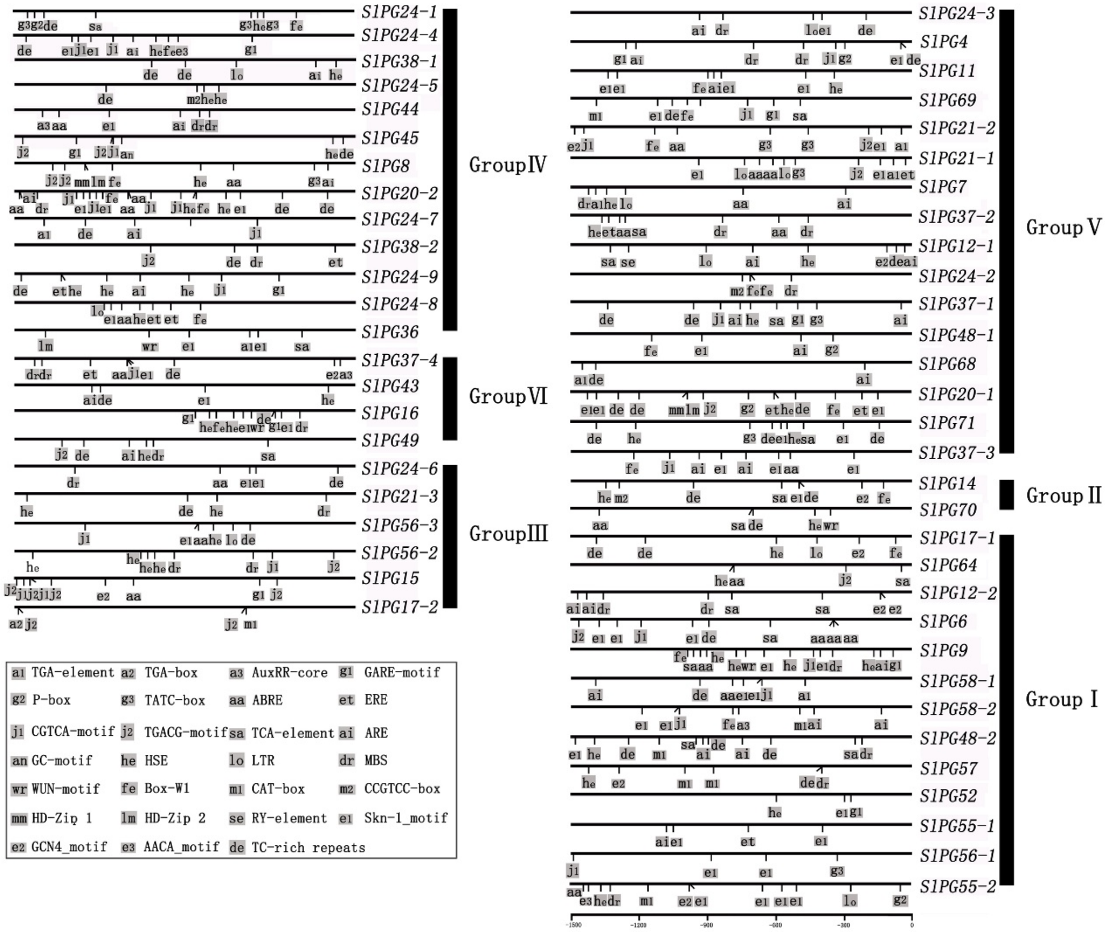

Figure S7. *Cis*-elements of plant hormones response, environmental stress response and specific organ/tissue development in promoter regions of the SlPG genes.

Table S2. The systematic names of PG genes in *Solanum lycopersicum*.

Arabidopsis	*Arabidopsis*	Tomato	Tomato gene ID	Identity
ATPG4	AT1G02460	SlPG4	Solyc01g079130.1.1	66.3
ATPG5	AT4G01890			
ATPG6	AT1G48100	SlPG6	Solyc04g008230.2.1	65.1
ATPG7	AT1G56710	SlPG7	Solyc01g100980.2.1	60.8
ATPG8	AT1G10640	SlPG8	Solyc07g015870.2.1	56.6
ATPG9	AT1G60590	SlPG9	Solyc05g005170.2.1	65.7
ATPG10	AT5G14650			
ATPG11	AT3G26610	SlPG11	Solyc05g005040.2.1	57.4
ATPG12	AT1G23460	SlPG12-1	Solyc09g098270.2.1	65.6
		SlPG12-2	Solyc04g025440.2.1	64.6
ATPG13	AT1G70500			
ATPG14	AT2G41850	SlPG14	Solyc10g080210.1.1	52
ATPG15	AT3G57510	SlPG15	Solyc04g015530.1.1	49.2
ATPG16	AT3G07970	SlPG16	Solyc01g087280.1.1	44.7
ATPG17	AT1G80170	SlPG17-1	Solyc03g116500.2.1	54.5
		SlPG17-2	Solyc06g068040.2.1	52.6
ATPG18	AT1G05650			
ATPG19	AT1G05660			
ATPG20	AT2G43890	SlPG20-1	Solyc08g014540.1.1	63.3
		SlPG20-2	Solyc12g019120.1.1	62.8
ATPG21	AT2G43880	SlPG21-1	Solyc12g019140.1.1	61.2
		SlPG21-2	Solyc12g019130.1.1	59.5
		SlPG21-3	Solyc08g014560.1.1	55.1
ATPG22	At2g43870			
ATPG23	AT3G59850			
ATPG24	AT1G65570	SlPG24-1	Solyc12g096740.1.1	49.1
		SlPG24-2	Solyc12g019220.1.1	48.9
		SlPG24-3	Solyc12g096730.1.1	48.9
		SlPG24-4	Solyc12g096750.1.1	48.3
		SlPG24-5	Solyc02g067650.1.1	48.3
		SlPG24-6	Solyc12g019180.1.1	48.1
		SlPG24-7	Solyc12g019230.1.1	47.3
		SlPG24-8	Solyc02g067630.2.1	46.9
		SlPG24-9	Solyc02g067640.2.1	46.9
ATPG25	AT2G43860			
ATPG26	AT2G15450			
ATPG27	AT2G15460			
ATPG28	AT2G15470			
ATPG29	AT2G26620			
ATPG30	AT2G40310			
ATPG31	AT4G13760			
ATPG32	AT1G43090			
ATPG33	AT1G43100			
ATPG34	AT1G43080			
ATPG35	AT1G17150			
ATPG36	AT1G78400	SlPG36	Solyc10g047570.1.1	40.8
ATPG37	AT2G33160	SlPG37-1	Solyc07g056290.1.1	49.1
		SlPG37-2	Solyc07g041650.1.1	44.2
		SlPG37-3	Solyc10g047590.1.1	40.8
		SlPG37-4	Solyc01g009640.1.1	39.1
ATPG38	AT3G07820	SlPG38-1	Solyc00g030510.2.1	47.8
		SlPG38-2	Solyc07g044870.2.1	47.3
ATPG39	AT3G07830			
ATPG40	AT3G07840			
ATPG41	AT5G48140			
ATPG42	AT3G07850			

Table S2 continued

Arabidopsis	*Arabidopsis*	Tomato	Tomato gene ID	Identity
ATPG43	AT3G14040	SlPG43	Solyc02g069480.1.1	42
ATPG44	AT4G18180	SlPG44	Solyc01g066070.2.1	40.5
ATPG45	AT1G02790	SlPG45	Solyc06g009200.2.1	41.7
ATPG46	AT3G06770			
ATPG47	AT5G49215			
ATPG48	AT3G16850	SlPG48-1	Solyc06g009790.2.1	57.1
		SlPG48-2	Solyc09g075460.2.1	56.3
ATPG49	AT4G23820	SlPG49	Solyc08g082170.2.1	70.7
ATPG50	AT5G41870			
ATPG51	AT3G62110			
ATPG52	AT4G33440	SlPG52	Solyc06g060170.2.1	64.4
ATPG53	AT2G23900			
ATPG54	AT3G48950			
ATPG55	AT3G61490	SlPG55-1	Solyc05g049980.2.1	71
		SlPG55-2	Solyc01g094970.2.1	66.7
ATPG56	AT4G23500	SlPG56-1	Solyc08g081480.2.1	69.2
		SlPG56-2	Solyc03g007940.2.1	63.8
		SlPG56-3	Solyc03g007950.2.1	61.9
ATPG57	AT1G19170	SlPG57	Solyc03g117750.2.1	68.8
ATPG58	AT3G42950	SlPG58-1	Solyc12g009210.1.1	72.9
		SlPG58-2	Solyc07g042160.2.1	57.4
ATPG59	AT3G57790			
ATPG60	AT5G44830			
ATPG61	AT5G44840			
ATPG62	AT4G35670			
ATPG63	AT5G27530			
ATPG64	AT4G32370	SlPG64	Solyc08g060970.2.1	39.2
ATPG65	AT4G32375			
ATPG66	AT4G32380			
ATPG67	AT3G15720			
ATPG68	AT5G17200	SlPG68	Solyc03g116580.2.1	40.4
ATPG69	AT5G39910	SlPG69	Solyc03g113230.1.1	38.6
ATPG70	AT1G80140	SlPG70	Solyc12g009420.1.1	35.7
ATPG71	AT4G20050	SlPG71	Solyc02g068400.2.1	62.4

Table S3. A summary of the number of SlPG genes belonged to different clades and groups in our phylogenetic and expression pattern analysis.

	Group Ⅰ	Group Ⅱ	GroupⅢ	GroupⅣ	Group Ⅴ	GroupⅥ	Total
**Clade A**	2	0	0	1	3	0	6
**Clade B**	2	1	2	0	1	1	7
**Clade C**	0	0	2	7	5	0	14
**Clade D**	0	0	0	5	3	2	10
**Clade E**	8	0	2	0	1	1	12
**Clade F**	1	1	0	0	2	0	4
**Clade G**	0	0	0	0	1	0	1
**Total**	13	2	6	13	16	4	54

Table S4. The putative *cis*-acting regulatory elements of plant hormones response, environmental stress response and specific organ/tissue development presented in the 5′-upstream region (1.5kb) of the polygalacturonase genes in *Solanum lycopersicum*.

Name	Putative *cis*-element	Sequence	Probable fuction
Plant hormones response related elements			
a1	TGA-element	AACGAC	auxin-responsive element
a2	TGA-box	TGACGTAA	part of an auxin-responsive element
a3	AuxRR-core	GGTCCAT	cis-acting regulatory element involved in auxin responsiveness
g1	GARE-motif	AAACAGA	gibberellin-responsive element
g2	P-box	CCTTTTG	gibberellin-responsive element
g3	TATC-box	TATCCCA	cis-acting element involved in gibberellin-responsiveness
aa	ABRE	TACGTG	cis-acting element involved in the abscisic acid responsiveness
et	ERE	ATTTCAAA	ethylene-responsive element
j1	CGTCA-motif	CGTCA	cis-acting regulatory element involved in the MeJA-responsiveness
j2	TGACG-motif	TGACG	cis-acting regulatory element involved in the MeJA-responsiveness
sa	TCA-element	CCATCTTTTT	cis-acting element involved in salicylic acid responsiveness
Environmental stress response related elements			
ai	ARE	TGGTTT	cis-acting regulatory element essential for the anaerobic induction
an	GC-motif	CCCCCG	enhancer-like element involved in anoxic specific inducibility
he	HSE	AAAAAATTTC	cis-acting element involved in heat stress responsiveness
lo	LTR	CCGAAA	cis-acting element involved in low-temperature responsiveness
dr	MBS	TAACTG	MYB binding site involved in drought-inducibility
de	TC-rich repeats	ATTTTCTCCA	cis-acting element involved in defense and stress responsiveness
wr	WUN-motif	TCATTACGAA	wound-responsive element
fe	Box-W1	TTGACC	fungal elicitor responsive element
Specific organ/tissue development related elements			
m1	CAT-box	GCCACT	cis-acting regulatory element related to meristem expression
m2	CCGTCC-box	CCGTCC	cis-acting regulatory element related to meristem specific
mm	HD-Zip 1	CAAT(A/T)ATTG	element involved in differentiation of the palisade mesophyll cells
lm	HD-Zip 2	CAAT(G/C)ATTG	element involved in the control of leaf morphology development
se	RY-element	CATGCATG	cis-acting regulatory element involved in seed-specific regulation
e1	Skn-1_motif	GTCAT	cis-acting regulatory element required for endosperm expression
e2	GCN4_motif	CAAGCCA	cis-regulatory element involved in endosperm expression
e3	AACA_motif	TAACAAACTCC	involved in endosperm-specific negative expression

Table S5. The types and number of putative *cis*-acting regulatory elements presented in the 5′-upstream region of the polygalacturonase genes in *S. lycopersicum*.

Gene	Plant Hormone Response Elements	Number	Environmental Stress Response Elements	Number	Specific Organ/Tissue Development Elements	Number
**Group IV**						
SlPG24-1	g2	1	de	1		
	g3	3	he	1		
	sa	1	fe	1		
SlPG24-4	g1	1	de	1	e1	2
	j1	2	he	1	e3	1
		1	fe	1		
			ai	1		
SlPG38-1			de	2		
			he	1		
			ai	1		
			lo	1		
SlPG24-5			de	1	m2	1
			he	2		
SlPG44	a3	1	ai	1	e1	1
	aa	1	dr	2		
SlPG45	j1	1	an	1		
	j2	2	he	1		
	g1	1	de	1		
SlPG8	j2	2	ai	1	mm	1
	g3	1	fe	1	lm	1
	aa	1	he	1		
SlPG20-2	aa	3	ai	1	e1	3
	j1	4	de	2		
			fe	2		
			he	2		
			dr	1		
SlPG24-7	a1	1	ai	1		
	j1	1	de	1		
SlPG38-2	j2	1	de	1		
	et	1	dr	1		
SlPG24-9	j1	1	ai	1		
	g1	1	de	3		
	et	1	he	1		
SlPG24-8	aa	1	lo	1	e1	1
	et	2	he	1		
			fe	1		
SlPG36	a1	1	wr	1	lm	1
	sa	1			e1	2
**Group VI**						
SlPG37-4	aa	1	de	1	e1	1
	a3	1	dr	2	e2	1
	j1	1				
SlPG43			ai	1	e1	1
			de	1		
			he	1		
SlPG16	g1	2	de	1	e1	2
			he	2		
			dr	1		
			fe	1		
			wr	1		
SlPG49	j2	1	ai	1		
	sa	1	de	1		
			he	1		
			dr	1		
**Group III**						
SlPG24-6	aa	1	de	1	e1	2
			dr	1		
SlPG21-3			de	1		

Table S5 continued

Gene	Plant hormone response elements	Number	Environmental stress response elements	Number	Specific organ/tissue development elements	Number
			he	2		
			dr	1		
SlPG56-3	aa	1	de	1	e1	1
	j1	1	he	1		
			lo	1		
SlPG56-2	j1	1	he	4		
	j2	1	dr	2		
SlPG15	j1	3			e2	1
	j2	4				
	aa	1				
SlPG17-2	a2	1			m1	1
	j2	2				
**Group V**						
SlPG24-3			ai	1	e1	1
			de	1		
			dr	1		
			lo	1		
SlPG4	j1	1	ai	1	e1	1
	g1	1	de	1		
	g2	1	dr	2		
SlPG11			ai	1	e1	4
			he	1		
			fe	1		
SlPG69	j1	1	de	1	e1	1
	g1	1	fe	1	m1	1
	sa	1				
SlPG21-2	aa	1	fe	1	e1	1
	a1	1			e2	1
	j1	1				
	j2	1				
	g3	2				
SlPG21-1	aa	2	lo	2	e1	2
	a1	1				
	et	1				
	j2	2				
SlPG7	aa	1	ai	1		
	a1	1	dr	1		
			he	1		
			lo	1		
SlPG37-2	aa	2	he	1		
	sa	1	dr	2		
	et	1				
SlPG12-1	sa	1	ai	2	e2	1
			de	1	se	1
			he	1		
			lo	1		
SlPG24-2			dr	1	m2	1
			fe	2		
SlPG37-1	j1	1	ai	2		
	g1	1	de	2		
	g3	1	he	1		
	sa	1				
SlPG48-1	g2	1	ai	1	e1	1
			fe	1		
SlPG68	a1	1	ai	1		
			de	1		
SlPG20-1	j2	1	de	3	e1	3
	g2	1	he	1	mm	1
	er	2	fe	1	lm	1
SlPG71	g3	1	de	3	e1	2
	sa	1	he	2		
SlPG37-3	ji	1	ai	2	e1	3

Table S5 continued

Gene	Plant hormone response elements	Number	Environmental stress response elements	Number	Specific organ/tissue development elements	Number
	aa	1	fe	1		
**Group II**						
SlPG14	sa	1	de	2	e1	1
			he	1	e2	1
			fe	1	m2	1
SlPG70	aa	1	de	1		
	sa	1	he	1		
			wr	1		
**Group I**						
SlPG17-1			de	2	e2	1
			he	1		
			fe	1		
			lo	1		
SlPG64	aa	1	he	1		
	j2	1				
	sa	1				
SlPG12-2	sa	2	ai	2	e2	2
			dr	2		
SlPG6	aa	3	de	1	e1	3
	j1	1				
	j2	1				
	sa	1				
SlPG9	aa	1	ai	1	e1	2
	j1	1	dr	1		
	g1	1	fe	1		
	sa	1	he	4		
			wr	1		
SlPG58-1	aa	1	ai	1	e1	2
	a1	1	de	1		
	j1	1				
SlPG58-2	a3	1	ai	2	e1	2
	j1	1	fe	1	ml	1
SlPG48-2	sa	2	ai	2	e1	1
			de	3	ml	1
			dr	1		
			he	1		
SlPG57			de	1	e2	1
			dr	1	ml	2
			he	1		
SlPG52	g1	1	he	1	e1	1
SlPG55-1	et	1	ai	1	e1	2
SlPG56-1	j1	1			e1	2
	g3	1				
SlPG55-2	aa	1	dr	1	e1	4
	g2	1	he	1	e2	1
			lo	1	e3	1
					ml	1

Table S6. Primers used in qRT-PCR analysis of PG genes in *Solanum lycopersicum*.

Gene Name	Primer Name	Primer Sequences (5′-3′)
SlPG4	SlPG4 Fp	TGTCTGTTTCAGATATTGAA
	SlPG4Rp	GCAAAAGTTCTATGTCTGAG
SlPG6	SlPG6 Fp	AAATGTTGAGATTGAAGCAG
	SlPG6 Rp	ATCGCGAATAATGGCGTTAC
SlPG7	SlPG7 Fp	TTACAGTGGAAGATTCAACT
	SlPG7 Rp	TGTTGTTGAAATTAATGTTG
SlPG8	SlPG8 Fp	GGCTGTTCAAGTTTATGGGA
	SlPG10 Rp	ATACAGTCATCTCCACAAGC
SlPG9	SlPG9 Fp	CTAAAGCCAATACAGGAGCG
	SlPG9 Rp	TGCTTGAATCTTGTTGCTCG
SlPG11	SlPG11 Fp	ACCAAACCAATTCATCTAGC
	SlPG11 Rp	GCAAGTTGATTAATTCCTCG
SlPG12-1	SlPG12-1 Fp	TATACTGTAACTCCGCCACG
	SlPG12-1 Rp	AACCTAAATCATAGTTCATC
SlPG12-2	SlPG12-2 Fp	CACAGGGATTGGTTATGGTT
	SlPG12-2 Rp	CGATGTATTCTTCCCTTGGT
SlPG14	SlPG14 Fp	TGCTCATGATTTTCAAGCTT
	SlPG14 Rp	TTTTCCATCACCCTTAGCTC
SlPG15	SlPG15 Fp	TCATTTTAAAGCTTATCCTT
	SlPG15 Rp	CTTCTTCATTGAATTTCTCC
SlPG16	SlPG16 Fp	TCATGGTCCTTGTAAGAAAG
	SlPG16 Rp	TGGAATAAAAGCCAATGTCT
SlPG17-1	SlPG17-1 Fp	TTCTCGTCCTATTCCAAATC
	SlPG17-1 Rp	GGTCCTCAAAGAAGCAGATG
SlPG17-2	SlPG17-2 Fp	CTTGGCAGGGAGGTAGAGGG
	SlPG17-2 Rp	TGATTTGGGCAGGGGAATGG
SlPG20-1	SlPG20-1 Fp	CTACCATTTATGTTCCACGT
	SlPG20-1 Rp	AGATGGAGCCACTAGGGTTC
SlPG20-2	SlPG20-2 Fp	GTAAAAGTAATAGCCCCAGA
	SlPG20-2 Rp	CTCCAGTTTTGATTCTACAA
SlPG21-1/ SlPG21-2	SlPG21-1/-2 Fp	TACAAGAATGTGAAAGGGAC
	SlPG21-1/-2 Rp	TCGTCTTAAACGATCGTTGT
SlPG21-3	SlPG21-3 Fp	AAAGGGGACATCATCAACAC
	SlPG21-3 Rp	TTTCAAACGAGGATCCACAA
SlPG24-1/ SlPG24-4	SlPG24-1/-4 Fp	TGTCATAACGCGATGATACA
	SlPG24-1/-4 Rp	CATAATACTAACTCCTGAGGAT
SlPG24-2	SlPG24-2 Fp	TGGTACTTTAGTGGCTCCCT
	SlPG24-2 Rp	CCAAAGATTAGCACCTTGAC
SlPG24-3	SlPG24-3 Fp	CGTTGCGATGATTGATGTTGA
	SlPG24-3 Rp	CACTTACTTTCACGCCCGAGA
SlPG24-5	SlPG24-5 Fp	TATACACGGAACATCGGCTAC
	SlPG24-5 Rp	GCTTTAGTCGGATGATCATTG
SlPG24-6	SlPG24-6 Fp	GGGAAGTTATCTATTGAACA
	SlPG24-6 Rp	ATTTAATCCAGTTACCAGAT
SlPG24-7	SlPG24-7 Fp	CAAGGAAGTTACTTGCTTAA
	SlPG24-7 Rp	ATTTGATCCAATTTTCTTCG
SlPG24-8/ SlPG24-9	SlPG24-8/-9 Fp	GGAACATCGGCTACAGAAGT
	SlPG24-8/-9 Rp	CTTCAGTCTGCTGATTTTGA
SlPG36	SlPG36 Fp	AATGGGGTGAGAATTAAGAG
	SlPG36 Rp	GATTATTATAGGATTCGAGG
SlPG37-1	SlPG37-1 Fp	CCATACAATGATTGTTCAAT
	SlPG37-1 Rp	AGAGGTTTATGTGGGAGTTC
SlPG37-2	SlPG37-2 Fp	TAACATAAGTGCACCACCCA
	SlPG37-3 Rp	CGCGATCATAGCAATACAAT
SlPG37-3	SlPG37-3 Fp	TCAGTTGCAGTTATGGATAT
	SlPG37-3 Rp	TACGGATGTTGTTTGAGTTG
SlPG37-4	SlPG37-4 Fp	GATTAAGACTTGGGCATCAT
	SlPG37-4 Rp	TAATATTGCAAGTATGTGAA
SlPG38-1	SlPG38-1 Fp	GATCAAGACATGGCCTGCTT
	SlPG38-1 Rp	TCTTTGTTGCATTTTCCACT
SlPG38-2	SlPG38-2 Fp	CAACAGGTCAGCCAGGAGTG
	SlPG38-2 Rp	TGATGCACATGCCTCTTTAA
SlPG43	SlPG43 Fp	TTCAACAGGAGATGATTGTG
	SlPG43 Rp	ACATCACCTTCATTTTGGTA
SlPG44	SlPG44Fp	AAGATAATACCTCCCACCCA
	SlPG44 Rp	GCGGAAGAGGGAATTAGAAC
SlPG45	SlPG45 Fp	GACCAGATCCCACTGCAGAT
	SlPG45 Rp	CCTGTAGTAAATGTTCCTGG
SlPG48-1	SlPG48-1 Fp	ATCTTACAACCCTTTTACAT
	SlPG48-1 Rp	GTGAAGCAAGTTGAAGTAGT
SlPG48-2	SlPG48-2 Fp	CATCAAAGATATTCTCATTT
	SlPG48-2 Rp	CCACTAACTGTAGGTAACAA
SlPG49	SlPG49 Fp	GATAGTACCAATGCAATATC
	SlPG49 Rp	TCAAGTGTTGAATTCTAAAG

Table S6 continued

Gene name	Primer name	Primer sequences (5′-3′)
SlPG52	SlPG52 Fp	AGGAGGAATAAGGAGACCGT
	SlPG52 Rp	ACACTAATCCTACCCGACCC
SlPG55-1	SlPG55-1 Fp	ATGGGATGCCAACAAGTCAA
	SlPG55-1 Rp	ATAGCCGTGATGTCCTCTGC
SlPG55-2	SlPG55-2 Fp	CATTTGATCATCAAACGTCT
	SlPG55-2 Rp	ATGGCTGTAATGTCTTGAAT
SlPG56-1	SlPG56-1 Fp	TTATCATCCAAGGCATCACTATCC
	SlPG56-1 Rp	CCACTCTTAACAGCAATACAATCA
SlPG56-2/ SlPG56-3	SlPG56-2/-3 Fp	ACAGCTCATTGTTCCTCCTG
	SlPG56-2/-3 Rp	TTAAATCCCACTCTGCCTCA
SlPG57	SlPG57 Fp	GCAACCTGACCCTACAGAAT
	SlPG57 Rp	CTCCTTGTCCACGAATACCA
SlPG58-1	SlPG58-1 Fp	CCCAAAGCTGTCCCATTACT
	SlPG58-1 Rp	AAGTGACGTTTCTCACCCAG
SlPG58-2	SlPG58-2 Fp	AAATCTAAAAGGGATTCAAG
	SlPG58-2 Rp	TACTTCTGATCTTCCCTCGT
SlPG64	SlPG64 Fp	CATTCATGTCAACCTCAGGT
	SlPG64 Rp	AACATTAGTGCAAGCTGTGC
SlPG68	SlPG68 Fp	TCAAATGCTTGCACCAATGA
	SlPG68Rp	ATCGTTACTCCCCTAACTCG
SlPG69	SlPG69 Fp	TGTTTTCTGAATGTGAAATT
	SlPG69 Rp	ATCCATTTTAAACATGCCAA
SlPG70	SlPG70 Fp	TGTCCGAGATGTCACGTTTG
	SlPG70 Rp	ACTCCAGTTCCCGTTTCCTT
SlPG71	SlPG71 Fp	AGGATTGTGGATTCTTATTT
	SlPG71 Rp	AACACCATTTATTGACTTCA
Ubi3	Ubi3Fp	TCCATCTCGTGCTCCGTCT
	Ubi3Rp	CTGAACCTTTCCAGTGTCAT
